# Clinical Telavancin Failure in Persistent Methicillin-Resistant Staphylococcus aureus Bacteremia

**DOI:** 10.7759/cureus.79588

**Published:** 2025-02-24

**Authors:** Kevin T Dao, Carlos D'Assumpcao, Rupam Sharma, Chandpreet Singh, Rasha Kuran, Arash Heidari

**Affiliations:** 1 Internal Medicine, UCLA-Kern Medical, Bakersfield, USA; 2 Infectious Diseases, UCLA-Kern Medical, Bakersfield, USA

**Keywords:** antibiotic resistance, cross-reactivity, evolution, immunocompromised, telavancin, vancomycin derivative

## Abstract

Telavancin is a fairly novel antibiotic derived from vancomycin developed to combat the constant evolutionary war against bacteria. It has achieved high clinical success in its role in treating Gram-positive organisms, although like in the case of any other antibiotics, failure can arise. The purpose of this article is to describe a case in which telavancin clinically failed in treating persistent methicillin-resistant *Staphylococcus aureus* (MRSA) bacteremia in a patient while describing the cause that led to telavancin failure.

## Introduction

For many years, bacteria have been able to develop resistance through various mechanisms to ensure survival. From efflux pumps to creating degradation enzymes that have prevented bacterial static or bactericidal events, unfortunately, constant evolution has shown bacteria to be quite resilient [1-2]. As such, a concerted effort is being made to develop new and potent antibiotics to keep up with the growing evolution of bacterial resistance. Telavancin is a fairly newly developed vancomycin-derivative semisynthetic lipoglycopeptide, designed to combat the evolving nature of methicillin-resistant bacteria [3-4]. Unfortunately, even with its success shown through prior clinical trials, failures can arise. Herein, we would like to describe a case where telavancin failed to clinically treat a patient with methicillin-resistant *Staphylococcus aureus* (MRSA) bacteremia and the subsequent measures taken to appropriately treat this patient’s bacteremia. Interestingly, this is one of the few cases that has been reported where telavancin has failed clinically.

## Case presentation

A 61-year-old man with a medical history of hypertension, type 2 diabetes mellitus, asthma, chronic obstructive pulmonary disease (COPD), depression, arthritis, and obstructive sleep apnea with a body mass index of 45 presented to the emergency department due to worsening shortness of breath. The patient had been wheelchair-bound nine years prior to presentation due to a car accident that resulted in a history of repeated scrotal abscesses and bacteremic MRSA infections based on prior chart review. The patient reported that he was unvaccinated against SARS-COV2 and had presented with a lack of taste and smell. A rapid polymerase chain reaction (PCR) test confirmed the patient to be SARS-COV2 positive, which resulted in the patient having more respiratory issues, predisposing him to use more steroids.

**Figure 1:**
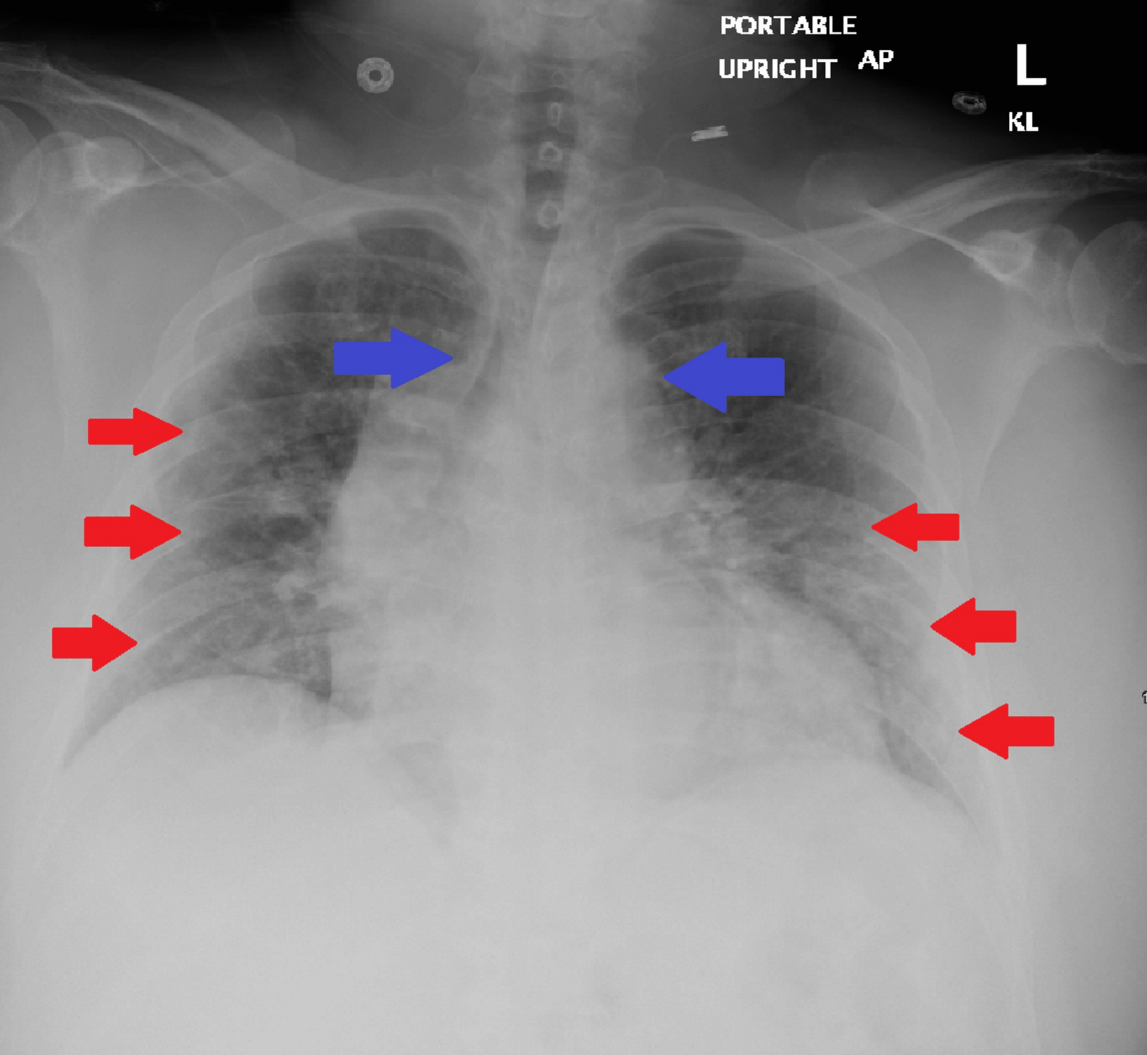
Chest X-ray AP: anteroposterior There is suboptimal inspiration with perihilar atelectasis and mild ground glass infiltrates which is worse on the left and highlighted by the red arrows which may represent suspected COVID-19 pneumonia. Of note, cardiomegaly is present with no large pulmonary effusions, edema, or pneumothorax. There is also mediastinal widening that is depicted by the blue arrows, but this may be due to vascular ectasia habitus, AP magnification, and/or possible other etiology. The patient's eosinophil sputum smear was also noted to be 6%

**Figure 2:**
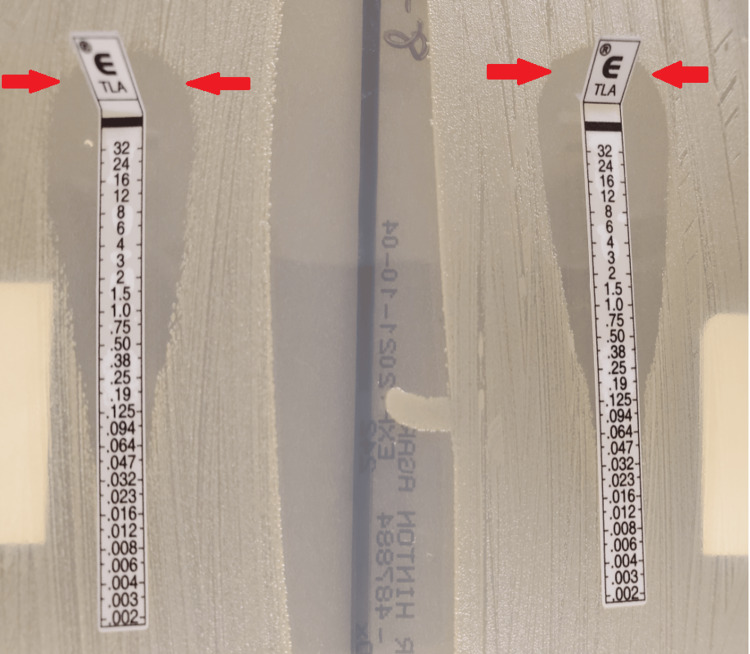
E test Telavancin E test results: on the left is hospital day 8 showing a minimum inhibitory concentration (MIC) of 0.064; however, on the right on day 21, the minimum inhibitory concentration had increased to 0.125. The arrows indicate the area where the antibiotic had prevented bacterial growth, and after hospital day 21, there is a noted decrease in width of how much telavancin was able to prevent bacterial growth

On physical examination, the patient’s initial vitals showed that the patient was febrile to 39.5, tachypneic to 34 breaths per minute, tachycardic to 115 beats per minute, and severely hypoxemic (Table 1). The patient was placed on six liters of oxygen via nasal cannula, and blood cultures, complete blood count, and basic metabolic panel were subsequently obtained (Tables 2-3). Initial imaging included a chest X-ray, which revealed significant perihilar atelectasis and mild ground glass infiltrates, which are worse on the left, possibly depicting COVID-19 pneumonia (Figure 1). Given a positive PCR result for SARS-COV2, the patient was initiated on two units of convalescent plasma, remdesivir 200 mg intravenous (IV) with 100 mg maintenance for five days, and dexamethasone 6 mg daily for ten days [5]. Blood cultures and nares screening obtained on arrival resulted positive for MRSA, and the patient was initially started on vancomycin and piperacillin-tazobactam. Blood cultures were repeated every 48 hours and remained positive with sensitivities showing susceptibility to vancomycin (Table 4). The patient was started on vancomycin with a 2000 mg bolus, with vancomycin 1500 mg every 12 hours with pharmacy dosing; however, after five days of persistent positive cultures, the decision was made to start telavancin 1000 mg IV daily, and an E test was done for both vancomycin (Table 4) and telavancin (Figure 2).

**Table 1:** Vitals

Vitals parameter	Recorded values on admission	Discharge values	Normal range
Systolic blood pressure	128 mmHg	120 mmHg	90-130 mmHg
Diastolic blood pressure	75 mmHg	78 mmHg	60-89 mmHg
Temperature	38.9°C	37.3 °C	34.4°C-38.3°C
Heart rate	100 beats/min	102 beats/min	60-100 beats/min
Respiratory rate	21 breaths/min	18 breaths/min	12-20 breath/min
Saturation of peripheral oxygen	83% (room air)	94 % (2 liter nasal cannula)	90%-100%

**Table 2:** Complete blood count

Parameter	Recorded values on admission	Discharge values	Normal range
White blood cell count	4.4 × 10^3^/mcL	7.9 × 10^3^/mcL	4.5-11 × 10^3^/mcL
Neutrophil absolute	3.6 × 10^3^/mcL	7.1 × 10^3^/mcL	1.8-7.7 × 10^3^/mcL
Band %	Not available	Not available	<12%
Lymphocyte absolute	0.5 × 10^3^/mcL	0.6 × 10^3^/mcL	1.2-4.5 × 10^3^/mcL
Absolute eosinophil count	0.3 × 10^3^/mcL	0 × 10^3^/mcL	<0.7 × 10^3^/mcL
Eosinophil %	0%	0%	<6%
Platelet count	274 × 10^3^/mcL	416 × 10^3^/mcL	150-450 x 10^3^/mcL

**Table 3:** Basic metabolic panel ALP: alkaline phosphatase

Parameter	Recorded values on admission	Discharge values	Normal range
ALP	99 units/L	156 units/L	45-117 units/L
Alanine aminotransferase	50 units/L	51 units/L	13-61 units/L
Aspartate aminotransferase	57 units/L	31 units/L	13-37 units/L
C-reactive protein	13.1 mg/dL	1.28 mg/dL	<0.30 mg/dL
Lactic acid	1.2 mmol/L	Not available	0.4-2 mmol/L
Lactate dehydrogenase	397 units/L	Not available	87-241 units/L
Procalcitonin	<0.10 ng/mL	<0.10 ng/mL	<0.10 ng/mL
Erythrocyte sedimentation rate	81 mm/hr	65 mm/hr	<20 mm/hr
Blood urea nitrogen	15 mg/dL	11 mg/dL	7-18 mg/dL
Creatinine	0.72 mg/dL	0.59 mg/dL	0.67-1.17 mg/dL

**Table 4:** Blood cultures and antibiotic with sensitivities n/a: not available; *HD: hospital day; **R: the resistance of the antibiotic; ***V1: Vitek 1; ****MIC: minimum inhibitory concentration; *****E test: E test also known as, “Epsilometer test”; +: the days the patient had received said antibiotic; +*: means beyond 29 hospital days and any + indicates that the patient received antibiotic beyond those days as indicated in the case report; TMP/SMX: trimethoprim-sulfamethoxazole

	HD* 9	HD 11	HD 12	HD 13	HD 16	HD 17	HD 19	HD 21	HD 23	HD 27	HD 29 +*
Oxacillin	R**	R	R	R	R	R	R	R	R	R	R
Vancomycin	V 1*** +	V 1	V 1	V 1	V 1	V 1	V 1	V 1	V 1	MIC**** < 0.5	V 1
	E test***** 1.5 +	n/a	n/a	n/a	n/a	n/a	n/a	E test 2	n/a	E test 2	n/a
Piperacillin-tazobactam	+	n/a	n/a	n/a	n/a	n/a	n/a	n/a	n/a	n/a	n/a
Telavancin	E test 0.064	+	+	+	+	+	+	E test 0.125 +	n/a	n/a	n/a
Daptomycin	E test 0.38	n/a	n/a	n/a	n/a	n/a	n/a	E test 0.75 +	+	E test 0.5	n/a
Ceftaroline	E test 0.25	n/a	n/a	n/a	n/a	n/a	n/a	E test 0.75 +	+	E test 0.5 +	+
Meropenem	n/a	n/a	n/a	n/a	n/a	n/a	n/a	n/a	+	+	+
Rifampin	n/a	n/a	n/a	n/a	n/a	n/a	n/a	n/a	n/a	MIC <0.5	+
Erythromycin	MIC <0.25	MIC < 0.25	MIC < 0.25	MIC < 0.25	MIC < 0.25	MIC < 0.25	MIC < 0.25	MIC < 0.25	MIC > 8	MIC < 0.25	MIC < 0.25
TMP/SMX	MIC <10	MIC < 10	MIC < 10	MIC < 10	MIC < 10	MIC < 10	MIC < 10	MIC < 10	MIC < 10	MIC < 10	MIC < 10

**Figure 3:**
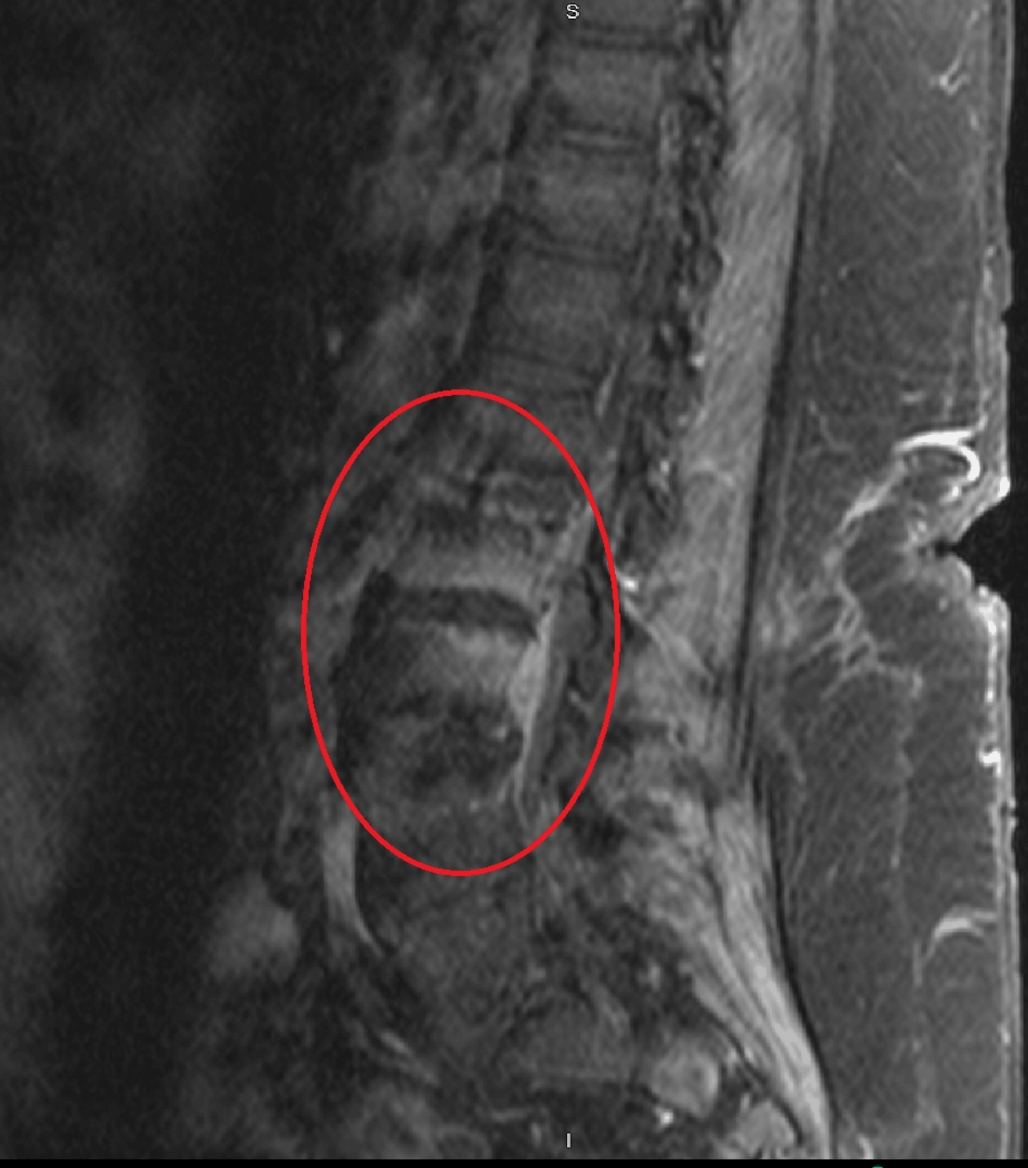
MRI spine lumbar w + wo contrast MRI: magnetic resonance imaging The red circle indicates spondylodiscitis and concerns of osteomyelitis, particularly severe degenerative disc disease at L1-L2 without posterior disc height and mild to moderate degenerative disc disease at L2-L3 with extensive vertebral body end plate edema

For several days, the patient stayed in the direct observation unit (DOU), and his respiratory status varied drastically to the point where the patient required a high-flow nasal cannula (HFNC) with forced inspiratory oxygen rate (FiO2) set at 55% and arterial blood gas (ABG) PaO2/FiO2 at 98. The patient also became tachycardic at 143 beats per minute, and oxygen saturation remained at 88% despite being placed on HFNC (Table 1). Fortunately, on hospital day 13, the patient’s vitals and clinical picture showed improvement, and he was downgraded to the medical floor. On hospital day 17, status post one week of telavancin treatment, the patient was endorsing new-onset lumbar back pain. Given this new finding, a concern was raised about osteomyelitis, and subsequently, magnetic resonance imaging (MRI) of the lumbar spine was ordered. Shortly after, interventional radiology (IR)-guided biopsy confirmed MRSA-induced osteomyelitis along with small phlegmons (Figure 3). A transesophageal echocardiogram (TEE) was also performed to rule out endocarditis, which resulted in inconclusive findings. On hospital day 21, a repeat E test showed that the patient’s bacterial infection was developing resistance to telavancin (Table 4). Consequently, telavancin was stopped after 11 days of treatment, and the patient was switched to ceftaroline 600 mg IV q12h, daptomycin 1 g IV, and nasal mupirocin twice daily with chlorhexidine mouthwash and soap [6]. E tests were also done on ceftaroline and daptomycin (Table 4). After several days of daptomycin treatment, the patient had increased dyspnea and sputum production. Analysis of sputum revealed eosinophilia, which was believed to be caused by daptomycin-induced eosinophilic pneumonitis (Table 2) [7].

The patient continued to be on a high-flow nasal cannula at 50 liters with FiO2 at 70%. Daptomycin was stopped, and ceftaroline was increased to q8h with meropenem added empirically for hospital-acquired pneumonia. A few days later, a repeated sputum Gram stain with culture was done, which was negative. The patient’s blood cultures on day 30 were also negative and continued to be negative until discharge. Meropenem was stopped, and 50 mg of prednisone was given daily for 2-4 months due to daptomycin-induced eosinophilic pneumonitis [7]. A TEE was also repeated and showed no valvular vegetation. The patient remained hemodynamically stable and was discharged in stable condition to a skilled nursing facility for continued physical therapy. His treatment course consisted of ceftaroline 600 mg IV q8h rifampin 300 mg PO q8 with repeated blood cultures for six weeks and MRSA decolonization for six months, approximately 40 days after hospital admission (Table 1), and was doing well at follow-up appointments.

## Discussion

Telavancin is a lipoglycopeptide antibiotic derived from vancomycin and causes depolarization of the cell membrane by binding to the D-ala-D-ala binding site, similar to vancomycin [8]. Due to vancomycin being a common antibiotic against Gram-positive organisms, bacteria tend to develop resistance by changing their membrane structure from D-ala-D-ala to D-lac (Figure 4) [9-10]. For this reason, telavancin was produced to rectify the upcoming resistance to vancomycin. Unfortunately, there have been some cases of cross-reactivity with telavancin and vancomycin in patients in certain HLA groups, but not among the general population [11]. Yet, cases of vancomycin-resistant organisms developing resistance to telavancin through cross-reactivity are few and far between. In fact, resistance to telavancin itself is not very common due to the novelty of the drug. Therefore, we would like to discuss why this is and how to prevent further resistance in the future.

On initial presentation, the patient had developed SARS-COV2 and had various comorbidities, particularly his use of steroids for asthma/COPD which made him quite susceptible [5,12]. It was hypothesized that this, in turn, allowed the bacterium to seed into various areas of the body; particularly the lumbar region, resulting in the production of phlegmons and osteomyelitis. It can be assumed that due to the fact that this patient had prior MRSA infections in the past, he became a host for the bacterium to slowly develop resistance (Figure 5). Prior cases have been documented regarding previous MRSA infections, which led to the colonization of subspecies with different mean inhibitory concentrations, resulting in MIC drift after prolonged antibiotic exposure (Figure 6) [1,2,13]. What is interesting is that this patient was unable to clear the infection despite being on telavancin, with sensitivities showing susceptibility and prior studies showing it to be quite a capable alternative (Table 4) [14]. After 11 days of telavancin and the patient continuing to grow positive blood cultures with a rising E test, it became clear that the bacterium was developing resistance possibly due to cross-reactivity since the patient had a history of frequent MRSA infections. The patient was also on vancomycin for five days, thus allowing the bug that was seeded in the lumbar regions of his spine to develop resistance because his osteomyelitis was never properly treated (Table 4) (Figure 2-3).

To rectify this issue, daptomycin along with either ceftaroline, trimethoprim-sulfamethoxazole (TMP-SMX), or fosfomycin were considered, since prior studies have shown this to be quite successful [6]. In this case, daptomycin and ceftaroline were the antibiotics of choice until the patient had to be switched due to an adverse reaction to daptomycin [7]. The main reason why telavancin was used was because this patient had an infection that was susceptible to telavancin and rather than use multiple drug treatments we believed it would be better to use one antibiotic that MRSA was susceptible to rather than many. Regardless, this multidrug regimen has shown success since the patient grew negative cultures shortly after and was stable enough to be discharged. This means that in certain cases where patients have recurrent infections with the same bacteria, using this antibiotic regimen should be considered [6].

In most instances, using one antibiotic with the sensitivities provided to treat an uncomplicated case of infection might be the ideal method. However, there have been situations where immunocompromised patients tend to have repeated infections, thereby allowing bacteria to evolve and develop antibiotic resistance [15-16]. With the advent of SARS-COV2, the use of steroids has become more prevalent, resulting in the possible rise of immunocompromised patients and bacterial resistance [12]. Therefore, in patients with multiple comorbidities and repeated infections, using multiple antibiotics should be heavily considered for clearing persistent infections.

## Conclusions

Overall, physicians should always be aware of the rapid evolution of bacteria, especially in immunocompromised patients with a history of repeated infections. It should be clear that resistance can still develop regardless of how novel the antibiotic is, especially if the said antibiotic is derived from another, in which resistance and cross-reactivity have been noted. Therefore, it would be wise to consider starting multiple antibiotics rather than one, regardless of E test and sensitivity results in patients who are more predisposed to infections and/or have many comorbidities. Of note, there are limitations to this case report. While this case does show a unique instance of bacterial evolution, cross-reactivity, persistence, and resistance, one case of failed telavancin should not mean that we should steer away from the antibiotic in complicated patients in all situations. Rather, it should be made clear that larger studies, clinical trials, etc., should be performed to validate this approach against persistent infections in complicated patients with various comorbidities. Antibiotic stewardship itself should also be key in using specific antibiotics against targeted bacteria once cultures and sensitivities are confirmed to prevent further resistance. Hopefully, this case report will spread awareness and provide insight into other cases on how to deal with the ongoing battle-clearing persistent infection.
